# What autism features in first episode psychosis? Results from a 2-year follow-up study

**DOI:** 10.1007/s00406-025-01986-1

**Published:** 2025-03-05

**Authors:** Lorenzo Pelizza, Antonio Federico, Emanuela Leuci, Emanuela Quattrone, Derna Palmisano, Simona Pupo, Giuseppina Paulillo, Clara Pellegrini, Pietro Pellegrini, Marco Menchetti

**Affiliations:** 1 Department of Biomedical and Neuromotor Sciences, Alma Mater Studiorum–Università di Bologna, Istituto di Psichiatria “Paolo Ottonello”, Via Pepoli, 5, 40126 Bologna, Italy; 2 Department of Mental Health and Pathological Addiction, Azienda USL di Parma, Parma, Province of Parma Italy; 3 Pain Therapy Service, Department of Medicine and Surgery, Azienda Ospedaliero-Universitaria di Parma, Parma, Province of Parma Italy

**Keywords:** PAUSS, Autism spectrum disorder, First episode psychosis, Psychopathology, Early intervention, Follow-up

## Abstract

The PANSS Autism Severity Score (PAUSS) is a popular measure of autistic features in First Episode Psychosis (FEP) samples. However, evidence on its longitudinal stability, course and treatment response is poor. Therefore, the main aim of this research was to compare clinical outcomes between FEP individuals with or without “autistic features” enrolled within an “Early Intervention in Psychosis” (EIP) service across 2 years of follow-up, as well as any significant association with EIP treatment components. FEP subjects completed the Positive And Negative Syndrome Scale (PANSS), the Global Assessment of Functioning (GAF), and the Health of the Nation Outcome Scale (HoNOS) at entry and across the follow-up. Statistical tests included Kaplan-Meyer survival analysis, mixed-design ANOVA, and multiple linear logistic regression analysis. 301 FEP subjects were enrolled (85 [28.0%] scored above the PAUSS cut-off score). Across the follow-up, the PAUSS + subgroup showed lower incidence rates of both symptomatic and functional remission. No PAUSS long-term stability was observed, but a statistically significant reduction in its values. This longitudinal change was mainly predicted by the total number of case management sessions offered within the EIP program. Our results suggest that the PAUSS could not represent a valid instrument to assess “trait-like” autistic features in FEP subjects. On contrary, it seems to capture a FEP subgroup characterized by higher severity levels in psychopathology and poorer outcomes and prognosis.

**Supplementary Information:**

The online version contains supplementary material available at 10.1007/s00406-025-01986-1.

## Introduction

*Autism features* are very common in patients with mental illnesses other than Autism Spectrum Disorders (ASD) [1], suggesting the idea of their transdiagnostic dimensionality [2]. This could represent the presence of *bio-behavioral phenotypes* rather than diagnostic entities, related to common neurodevelopmental pathways and modulating biological risk factors [3, 4].

In this respect, autism and *psychosis*, which were historically considered to be closely associated [5, 6], co-occur at elevated rates [7]. Recent epidemiological reviews on individuals with psychosis reported heterogeneity in prevalence rates of ASD-like characteristics at both the trait level (10–60%) and the diagnostic level (1–50%) [8–11]. In particular, the multiform category of psychosis seems include a distinct *subgroup* with relevant autistic features [12]: i.e., an *autistic phenotype* typically characterized by marked impairment in emotion elaboration, communication, and social interaction [13]. This subtype appears to be associated with longer duration of illness, earlier age at onset, greater severity in psychopathology, more severe cognitive deficits, higher risk of suicide, and poorer global functioning [14–17]. In particular, patients with prominent *negative features* often showed higher scores on ADS scales, supporting the idea of similar underlying neurodevelopmental vulnerability and overlapping biological pathways [18].

Given their negative impact on outcomes and prognosis, research and clinical interest has recently focused on autism characteristics in early psychosis (especially in *First Episode Psychosis* [FEP]) [19], also due to the development of a new, quick psychopathological dimension to measure autistic features using 8 specific items of the Positive And Negative Syndrome Scale (PANSS): i.e., the “PANSS Autism Severity Score” (*PAUSS*) [20]. The PAUSS items were selected to cover the 3 main ASD domains as defined in the DSM-IV-TR criteria (i.e., repetitive/stereotyped patterns of behavior and difficulties in communication and social interaction) [21] and showed good convergent validity with ASD measures [20, 22] and good internal consistency promoting their aggregation into a continuous autism severity score [20, 23]. Specifically, it has been reported that young patients with FEP highly scoring on the PAUSS have lower educational levels, greater severity in psychopathology (especially in negative symptoms), higher cognitive deficits, and poorer daily functioning [24–26].

Although the PAUSS remains a popular scale to evaluate autism characteristics in psychosis [27], evidence on its longitudinal course is very poor, as well as on its treatment response, especially in youths with FEP. This may severely prejudice its clinical meaning, also taking into account the recent results reported in a longitudinal investigation [28] showing a fair long-term stability of the PAUSS. According to the authors, this finding suggests that the PAUSS could *not capture autistic features*, but represent a proxy for clinical severity in psychotic-state symptoms (especially the negative ones) [29]. Differently, Jeong and collegues [22] reported a persistence of the PAUSS autistic features over 2 years of follow-up, suggesting that they could represent enduring attributes rather than a simple state variable.

Starting from this background, the *aims* of this examination were: (1) to compare baseline clinical and sociodemographic parameters between FEP patents with or without autistic characteristics recruited within an “Early Intervention in Psychosis” (EIP) service; (2) to investigate any inter-group difference in clinical outcomes across a 2-year follow-up period; and (3) to examine longitudinal stability of the PAUSS in both the FEP total group and the subgroup with autistic features, as well as any significant relationships with treatment components provided within the EIP program. Specifically, we examined the PAUSS longitudinal course in order to analyze whether it remained stable over time or whether its longitudinal change was associated with a putative treatment response.

## Methods

### Setting

Participants were FEP inpatients or outpatients consecutively enrolled within the “Parma-Early Psychosis” (*Pr-EP*) protocol between January 2013 and December 2022. The Pr-EP program is a diffused EIP infrastructure implemented in all adolescent and adult services of the Parma Department of Mental Health, in Northern Italy [30, 31].

For the specific goals of this investigation, *inclusion criteria* were: (a) age 12–35 years; (b) specialist help-seeking need; (c) baseline DSM-5 diagnoses of schizophrenia, schizophreniform disorder, bipolar disorder/major depressive disorder with psychotic features, brief psychotic disorder, delusional disorder, and psychotic disorder not otherwise specified [32]; and (d) a DUP (“Duration of Untreated Psychosis”) [33] of < 2 years. This DUP was specifically selected because it is considered the usual time limit to offer effective treatments within the EIP paradigm [34, 35].

*Exclusion criteria* were: (a) previous DSM-5 psychotic episode; (b) previous exposure to antipsychotic (AP) drug; (c) known intellectual disability (i.e., IQ < 70); and (d) medical/neurological disease manifesting with psychiatric symptoms. Past AP exposure (i.e., before the Pr-EP recruitment) was considered a “functional equivalent” of past psychotic episode, as originally defined in the criteria for psychosis threshold within the EIP paradigm (i.e., “…those patients for whom AP drug would probably be started in clinical practice”) [36, 37]. Patients with comorbid substance abuse at entry were included in the study.

### Assessment

In this examination, psychopathological evaluation included the PANSS [38], the GAF (“Global Assessment of Functioning” scale) [32], and the HoNOS (“Health of the Nation Outcome Scale”) [39]. They were completed by trained Pr-EP team members both at baseline and every year during the follow-up. Scoring workshops and supervision sessions were regularly performed to ensure inter-rater reliability [40].

The *PANSS* is a clinical interview commonly used to evaluate psychopathology in psychosis, including FEP [41, 42]. The *PAUSS* included the following 8 PANSS items covering the 3 main DSM-IV-TR ASD domains [21]: N1 “Blunted affect”, N3 “Poor rapport”, N4 “Passive/apathetic social withdrawal”, N5 “Difficulty in abstract thinking”, N6 “Lack of spontaneity and flow of conversation”, N7 “Stereotyped thinking”, G5 “Mannerisms and posturing”, and G15 “Preoccupation” (see Supplementary Materials [Table S1] for details). Greater total scores on the PAUSS denoted higher severity levels of autism characteristics. Specifically, in accordance with Kastner and co-workers [20], FEP individuals scoring above 30 were considered as having clinically relevant autistic features. Furthermore, as proposed by Shafer and Dazzi [43], we considered the following 5 main PANSS dimensions to explore psychosis psychopathology: “Disorganization”, “Negative Symptoms”, “Positive Symptoms”, “Affect” (“Depression/Anxiety”), and “Resistance/Excitement-Activity”. Finally, as index of symptomatic remission at follow-ups, we considered a score of ≤ 3 (corresponding to mild severity or less) on all the 8 PANSS items indicated in the Remission in Schizophrenia Working Group’s criteria [44].

The *GAF* is a widely used scale developed to evaluate clinical and socio-occupational functioning in individuals with mental disorders [32], including psychosis [45]. In our outcome analysis, we considered a GAF score of ≥ 60 at follow-ups as current index of functional remission in accordance with what was proposed by Yang and co-workers [46].

The *HoNOS* is a commonly used instrument specifically developed to assess mental health and social outcomes in subjects with severe mental illness, including psychosis [47]. As indicated by Morosini and his team [48], we examined 4 main outcome dimensions: “Impairment”, “Psychiatric Symptoms”, “Behavioral Problems”, and “Social Problems”. In our outcome examination, we also considered HoNOS item 9, 10, and 11 scores of ≤ 2 at follow-ups as a current index of functional remission in accordance with what was indicated by Kortrijk and colleagues [49].

Lastly, a clinical/sociodemographic chart was also completed at baseline and at follow-ups, also including information on DUP, service disengagement, new suicide attempt, new hospitalization, current suicidal ideation, functional recovery, and Pr-EP pharmacological and psychosocial interventions [50]. Specifically, “*suicide attempt*” was defined as potentially injurious, self-inflicted behavior without a fatal outcome for which there was (implicit or explicit) evidence of intent to die [51, 52]. *Service disengagement* was described as complete lack of contact or untraceable for at least 3 months despite a need of treatment [47, 53]. Current *suicidal ideation* was detected as a score of ≥ 2 on item 4 (“Suicidality”) of the Brief Psychiatric Rating Scale (BPRS), corresponding at least to occasional suicidal thinking without specific plans [54]. Finally, in our outcome analysis, functional recovery was also simply defined as “return to work/school”, as proposed by Silva and Restrepo [55].

The *DSM-5 diagnosis* was identified at presentation by two trained Pr-EP team professionals using the Structured Clinical Interview for DSM-5 mental disorders (SCID-5) [56]. The presence of FEP was also formulated using the psychometric threshold as defined in the Comprehensive Assessment of At-Risk Mental States (CAARMS), authorized Italian version [57, 58].

### Procedures

At entry, FEP individuals were divided into two subsamples based on PAUSS cut-off score of > 30 (i.e., *FEP/PAUSS* + vs FEP/PAUSS-) [20]. In this respect, the last percentile of our PAUSS distribution corresponded to the same score value reported by Kastner and colleagues [20].

Inter-group comparisons on clinical, sociodemographic and treatment parameters at presentation were first examined. Across the 2-year follow-up period, the two subgroups were compared in terms of clinical outcomes (e.g., service disengagement, new hospital admission, new suicide attempt) and stability of GAF, HoNOS, and PANSS scores. Between-group comparisons on treatment response were also explored. In this respect, the Pr-EP program is a specialized EIP protocol offering a 2-year comprehensive intervention package including a psychopharmacological treatment and a multi-component psychosocial intervention, which specifically combines individual psychotherapy (based on the cognitive-behavioral model), psychoeducational sessions for family members, and a recovery-oriented case management [59], in line with the current EIP guidelines [60, 61].

### Statistical analysis

Collected data were analyzed using the Statistical Package for Social Science (SPSS) 15.0 for Windows [62]. Significance level was set at 0.05 for all two-tailed tests. In inter-group analyses, Chi-square (Χ^2^) or Fisher exact test (as appropriate) for categorical variables [63] and Mann–Whitney U test for continuous variables were performed, using the Bonferroni p correction [64].

As for time-to-event outcome measures in our longitudinal examinations (e.g., service disengagement, new suicide attempt), Kaplan–Meier survival analyses were carried out, while for not time-to-event dependent measures (e.g., current suicidal ideation, functional recovery) binary logistic regression analyses with PAUSS groups as independent parameter were performed [65].

A mixed-design ANOVA was also used to examine inter-group comparisons on longitudinal course and stability of psychopathological and outcome parameters (i.e., PANSS, GAF, and HoNOS scores). Furthermore, the Wilcoxon test for repeated measures was also carried out to investigate the longitudinal stability of PAUSS scores over time. Finally, a multiple linear regression analysis was performed to explore any relevant association between longitudinal change in PAUSS score (as dependent parameter) and intensity of Pr-EP intervention components offered across the 2 years of follow-up (as independent parameters). In this examination, we specifically used the difference (delta [Δ]) between T0 and T2 PAUSS scores as primary psychopathological variable to investigate overtime. Indeed, in line with what was reported by Ver Hoef [66], the delta score better describes longitudinal changes and temporal dynamics of psychopathology compared to T0 and T2 single subscores.

## Results

In this investigation, 301 FEP individuals (195 [64.8%] males; mean age = 25.05 ± 7.19 years) were recruited: of them, 85 (28.2%) scored above the PAUSS cut-off score of 30 and were included in the FEP/PAUSS + subgroup. The remaining 216 participants were grouped in the FEP/PAUSS- subsample. Clinical and sociodemographic characteristics of the two groups are show in the Table 1.

**Table 1:** Sociodemographic and clinical comparisons in the two FEP subgroups at baseline (n = 301)

Variable	FEP/PAUSS- (n = 216)	FEP/PAUSS + (n = 85)	X2	p
Gender (males)	140 (64.8%)	55 (64.7%)	.001	.986
Citizenship (Italian)	182 (84.3%)	71 (83.5%)	.024	.876
Ethnicity			8.970	.089
White	182 (84.3%)	71 (83.5%)		
Black	4 (1.9%)	5 (5.9%)		
Asian	7 (3.2%)	6 (7.1%)		
North African	14 (6.5%)	1 (1.2%)		
Other ethnic group^a^	9 (4.2%)	2 (2.4%)		
Migrant Status	44 (20.4%)	24 (28.3%)	2.158	.142
Age (at entry)	24.82 ± 6.99	25.61 ± 7.70	− .560	.576
Education (in years)	11.72 ± 2.59	11.89 ± 2.92	− .294	.769
Civil status			.469	.926
Single	196 (90.7%)	77 (90.6%)		
Married/partnership	16 (7.4%)	7 (8.2%)		
Separated/divorced	4 (1.9%)	1 (1.2%)		
Living status				
Alone	13 (6.0%)	4 (4.7%)	2.052	.562
Living with partners	23 (10.6%)	8 (9.4%)		
Living with parents	167 (77.3%)	64 (75.3%)		
Other cohabitation	13 (6.0%)	9 (10.6%)		
Occupation			2.387	.303
NEET	112 (51.9%)	47 (55.3%)		
Student	54 (25.0%)	25 (29.4%)		
Employed	50 (23.1%)	13 (15.3%)		
Source of referral			2.898	.716
Primary care	59 (27.3%)	22 (25.9%)		
Family members	29 (13.4%)	10 (11.8%)		
Self-referral	16 (7.4%)	8 (9.4%)		
Emergency room	80 (37.0%)	30 (35.3%)		
School/Social services	5 (2.3%)	5 (5.9%)		
Other mental health services	27 (12.5%)	10 (11.8%)		
DUP (in weeks)	42.59 ± 44.25	41.87 ± 42.69	− .455	.649
Family history for psychosis	81 (37.5%)	27 (31.7%)	.240	.802
Baseline hospitalization	103 (47.7%)	46 (54.1%)	1.010	.315
Baseline compulsory admission	28 (13.0%)	10 (11.8%)	.079	.778
Previous specialist contact	102 (47.2%)	39 (45.9%)	.044	.834
Previous suicide attempt	15 (6.9%)	10 (11.8%)	1.861	.173
Substance misuse (at entry)	93 (43.1%)	32 (37.6%)	.735	.391
Treatments				
Baseline AP prescription	192 (88.9%)	78 (98.1%)	.546	.460
Baseline AP LAI prescription	26 (12.0%)	11 (12.9%)	.015	.903
Baseline equivalent dose of risperidone (mg/day)	4.00 ± 3.06	3.78 ± 2.24	− .466	.655
Baseline AD prescription	34 (15.7%)	17 (20.0%)	.786	.375
Baseline equivalent dose of fluoxetine (mg/day)	35.18 ± 15.65	26.47 ± 11.69	− .2.599	**.010**
Baseline MS prescription	29 (13.4%)	11 (12.9%)	.012	.911
Baseline BDZ prescription	86 (39.8%)	28 (32.9%)	1.225	.268
Baseline equivalent dose of lorazepam (mg/day)	3.24 ± 2.19	2.54 ± 1.70	− 1.348	.178
Baseline acceptance of individual psychotherapy	162 (75.0%)	72 (84.7%)	3.321	.068
Baseline acceptance of family psychoeducation	134 (62.0%)	61 (71.8%)	2.530	.112
Baseline acceptance of case management	143 (62.6%)	74 (87.1%)	13.186	**.001**
DSM-5 diagnosis			21.647	**.001**
Schizophrenia	92 (42.6%)	34 (40.0%)	.168	.681
Affective psychosis	44 (20.4%)	13 (15.3%)	1.024	.312
Brief psychotic disorder	27 (12.5%)	9 (10.6%)	.212	.645
Psychotic disorder NOS	31 (14.4%)	7 (8.2%)	2.069	.150
Schizoaffective disorder	8 (3.7%)	1 (1.2%)	1.343	.453
Schizophreniform disorder	14 (6.5%)	21 (24.7%)	19.715	**.001**
Schizophrenia spectrum disorder^b^	114 (52.8%)	56 (65.9%)	4.262	**.038**
PANSS scores				
Positive Symptoms	17.77 ± 6.00	18.38 ± 6.54	− .619	.536
Negative Symptoms	19.55 ± 6.24	31.74 ± 5.64	− 11.755	**.001**
Disorganization	17.86 ± 5.32	28.92 ± 6.00	− 11.393	**.001**
Affect	15.87 ± 5.36	16.93 ± 5.84	− 1.530	.126
Resistance/Excitement-activity	9.44 ± 4.63	12.51 ± 5.12	− 4.883	**.001**
PANSS total score	83.92 ± 19.44	111.75 ± 21.69	− 8.819	**.001**
G12 “Lack of judgment/insight”	3.40 ± 1.97	3.28 ± 1.98	.465	.642
P7 “Hostility”	2.79 ± 1.68	3.05 ± 1.74	− 1.219	.223
G8 “Uncooperativeness”	2.38 ± 1.64	3.07 ± 1.94	− 2.945	**.003**
P4 “Excitement”	1.75 ± 1.45	1.80 ± 1.40	− .890	.373
G14 “Poor impulse control”	2.55 ± 2.04	4.59 ± 2.38	− 6.516	**.001**
GAF score	44.34 ± 10.07	43.18 ± 11.40	.818	.413
HoNOS score				
Behavioral problems	3.49 ± 2.36	4.27 ± 2.57	− 2.237	**.025**
Impairment	2.01 ± 1.48	4.36 ± 1.88	− 8.983	**.001**
Psychiatric symptoms	10.59 ± 3.72	11.23 ± 2.80	− .838	.402
Social problems	6.21 ± 3.14	9.92 ± 3.73	− 6.952	**.001**
“Overactive/aggressive/disruptive/agitated behavior”	0.45 ± 0.80	0.50 ± 0.94	− 3.489	**.001**
“Non-accidental self-injury”	0.24 ± 0.66	0.11 ± 0.31	1.441	.150
“Problem drinking/drug-taking”	0.46 ± 0.91	0.42 ± 0.95	.645	.513
“Cognitive problems”	0.80 ± 0.87	1.43 ± 0.91	− 8.670	**.001**
“Physical illness or disability problems”	0.42 ± 0.68	0.51 ± 0.81	− .155	.122
“Problems with relationships”	1.27 ± 1.06	1.74 ± 0.84	− 5.680	**.001**
“Problems with activities of daily living”	0.96 ± 1.00	1.46 ± 0.78	− 6.618	**.001**
“Problems with housing and living conditions”	0.50 ± 0.83	1.15 ± 0.92	− 4.437	**.001**
“Problems with occupation and activities”	0.46 ± 0.71	1.00 ± 0.89	− 4.799	**.001**
Antipsychotic presciption	190 (88.0%)	74 (87.1%)	.046	.830
Antidepressant prescription	34 (15.7%)	17 (20.0%)	.786	.375
Mood stabilizer prescription	29 (13.4%)	11 (12.9%)	.012	.911
Benzodiazepine prescription	86 (39.8%)	28 (32.9%)	1.225	.268

Frequencies (and percentages) and mean ± standard deviation are reported. Chi-square (X2) test (or Fisher exact test, as appropriate) and Mann–Whitney (z) test values are reported. Bonferroni’s corrected p values are reported. Statistically significant p values are in bold

*FEP* First episode psychosis, *PAUSS* PANSS autism severity scores, *FEP/PAUSS +* FEP patients with PAUSS score of > 30, *FEP/PAUSS-* FEP patients without PAUSS score of > 30, *NEET* Not in Education, Employment, nor Training, *DUP* Duration of untreated psychosis, *AP* Antipsychotic medication, *AP LAI* Antipsychotic long-acting injection, *AD* Antidepressant medication, *MS* Mood Stabilizer, *BDZ* Benzodiazepine, *DSM-5* Diagnostic and Statistical Manual of mental disorders, 5th Edition, *PANSS* Positive and negative syndrome scale, *GAF* Global assessment of functioning, *HoNOS* Health of the nation outcome scale.

aOther ethnicity included Hispanic and other ethnic group; bSchizophrenia spectrum disorder included schizophrenia, schizophreniform disorder, and schizoaffective disorder

### Baseline comparisons

Compared to FEP/PAUSS-, FEP/PAUSS + participants showed lower equivalent dose of fluoxetine, higher prevalence rates of case management and Schizophrenia Spectrum Disorder (SSD) diagnosis (especially schizophreniform disorder), greater PANSS total and “Negative Symptoms”, “Disorganization”, and “Resistance/Excitement-Activity” dimension scores (especially in terms of “Uncooperativeness” and “Poor Impulse Control” item subscores). Moreover, they had greater HoNOS “Behavioral Problems”, “Impairment”, and “Social Problems” domain scores (especially in terms of “Overactive/Aggressive/Disruptive/Agitated Behavior”, “Cognitive Problems”, “Problems with Relationships”, “Problems with Activities of Daily Living”, “Problems with Housing/Living Conditions”, and “Problems with Occupation/Activities” item subscores) (Table 1).

### Longitudinal comparisons

In comparison with FEP/PAUSS-, FEP/PAUSS + subjects showed lower 1-year incidence rates of both HoNOS functional remission and PANSS symptomatic remission. Moreover, they had lower 2-year incidence rates of service disengagement (Table 2) (see also Supplementary Materials [Figure S1] for details on Kaplan-Meyer survival functions) and PANSS symptomatic remission (Table 3).

**Table 2:** Kaplan–Meier survival analysis results: comparisons on 2-year time-to-event outcome incidence rate among the two FEP subgroups

FEP subgroup	Number of events	cumulative proportion surviving at the time		Mean (in months) for 2-year service disengagement incidence rate			
		Estimate	SE	Estimate	SE	95% CI	
						Lower bound	Upper bound
FEP/PAUSS + FEP/PAUSS- (Overall)	5 (5.9%) 67 (31.0%) 72 (23.9%)	.941 .690 –	.026 .031 –	23.165 19.981 20.880	.382 .427 .335	22.417 19.145 20.224	23.912 20.818 21.537
Log Rank (Mantel-Cox)				Χ2	df	p	
				19.647	1	**.001**	
FEP subgroup	Number of events	cumulative proportion surviving at the time		Mean (in months) for 2-year new hospitalization rate			
		Estimate	SE	Estimate	SE	95% CI	
						Lower bound	Upper bound
FEP/PAUSS + FEP-PAUSS- (Overall)	26 (30.5%) 56 (25.9%) 82 (27.2%)	.682 .698 –	.052 .034 –	20.528 20.768 20.697	.576 .368 .310	19.400 20.047 20.090	21.656 21.489 21.305
Log Rank (Mantel-Cox)				Χ2	df	p	
				.088	1	.767	
FEP subgroup	Number of events	cumulative proportion surviving at the time		Mean (in months) for 2-year new suicide attempt incidence rate			
		Estimate	SE	Estimate	SE	95% CI	
						Lower bound	Upper bound
FEP/PAUSS + FEP/PAUSS- (Overall)	4 (4.7%) 12 (5.5%) 16 (5.3%)	.951 .935 –	.024 .018 –	23.475 23.323 23.370	.261 .191 .155	22.962 22.948 23.066	23.987 23.698 23.673
Log Rank (Mantel-Cox)				Χ2	df	p	
				.250	1	.617	

Significant statistical p values are in bold

*FEP* First episode psychosis, *PAUSS* PANSS Autism severity scores, *FEP/PAUSS +* FEP patients with PAUSS score of > 30, *FEP/PAUSS-* FEP patients without PAUSS score of > 30, *SE* Standard error, *95% CI* 95% Confidence intervals; *Log Rank* Logarithm rank test, *X2* Chi-Square test, *df* degrees of freedom; *p* statistical value

**Table 3:** Binary logistic regression analysis results for 2-year not time-to-event outcome variables in the two FEP subgroups

Dependent variable (n = 276)	FEP/PAUSS- (n = 195)	FEP/PAUSS + (n = 81)	Statistic test				
			B (SE)	HR	95% CI		p
					Lower Higher		
1-year current suicidal ideation 1-year functional recovery 1-year GAF functional remission 1-year HoNOS functional remission 1-year PANSS symptomatic remission	45 (23.1%) 105 (53.8%) 102 (52.3%) 144 (73.8%) 98 (50.3%)	27 (33.3%) 39 (48.1%) 37 (45.7%) 46 (56.8%) 10 (12.3%)	.515 (.285) − .108 (.257) − .168 (.192) .745 (.265) 1.885 (.363)	.597 .897 1.182 2.106 6.587	.341 .543 .714 1.254 3.232	1.045 1.483 1.960 3.537 13.423	.071 .672 .515 **.005** **.001**
Dependent variable (n = 229)	FEP/PAUSS- (n = 150)	FEP/PAUSS + (n = 79)	Statistic test				
			B (SE)	HR	95% CI		p
					Lower Higher		
2-year current suicidal ideation 2-year functional recovery 2-year GAF functional remission 2-year HoNOS functional remission 2-year PANSS symptomatic remission	23 (15.3%) 115 (76.7%) 99 (66.0%) 129 (86.0%) 99 (66.0%)	10 (12.7%) 11 (13.9%) 51 (64.6%) 65 (82.3%) 28 (35.4%)	.297 (.371) .632 (.378) -.275 (.264) -.140 (.310) .862 (.267)	1.345 1.881 .759 .869 2.369	.650 .897 .453 .473 1.405	2.785 3.947 1.274 1.596 3.994	.424 .095 .297 .651 **.001**

Significant statistical p values are in bold. Cumulative incidence rates are reported. Current suicidal ideation = BPRS item 4 score of ≥ 2; Functional recovery = return to work/school; GAF functional remission = GAF score of ≥ 60; HoNOS functional remission = HoNOS item 9, 10, and 11scores of ≤ 2; PANSS symptomatic remission = PANSS item P1, P2, P3, N1, N4, N6, G5, G9 scores of ≤ 3

*FEP* First episode psychosis, *PAUSS* PANSS Autism severity scores, *FEP/PAUSS + * FEP patients with PAUSS score of > 30, *FEP/PAUSS-* FEP patients without PAUSS score of > 30, *GAF* Global assessment of functioning, *HoNOS* Health of the nation outcome scale, *PANSS* Positive and negative syndrome scale, *B* regression coefficient, *SE* Standard error, *HR* Hazard ratio, *95% CI* 95% confidence intervals for HR, *p* statistical significance

Our mixed-design ANOVA results showed a longitudinal improvement in all GAF, HoNOS, and PANSS scores (Table 4). However, there was evidence of statistically relevant “group effects” for all PANSS dimension subscores, PANSS total score, GAF score, and HoNOS “Impairment”, “Psychiatric Symptoms”, and “Social Problems” domain subscores. Specifically, compared to FEP/PAUSS-, our FEP/PAUSS + individuals showed lower GAF scores and greater PANSS and HoNOS scores over time (see Supplementary Materials [Figure S2] for details on profile plots).

**Table 4:** Mixed-design ANOVA results: psychopathological and outcome parameters across the 2-year follow-up period in the two FEP subgroups

Variable	Time effect				Group effect				Interaction effect (time × group)			
	df	F	p	η2	df	F	p	η2	df	F	p	η2
PANSS Positive symptoms	1.6	198.017	**.001**	.466	1	5.887	**.016**	.025	1.6	1.909	.158	.008
PANSS Negative symptoms	1.8	161.696	**.001**	.417	1	142.495	**.001**	.387	1.8	19.612	**.001**	.080
PANSS Disorganization	1.6	265.759	**.001**	.542	1	149.181	**.001**	.399	1.6	36.343	**.001**	.139
PANSS Affect	1.7	169.233	**.001**	.427	1	6.540	**.011**	.028	1.7	.958	.372	.004
PANSS Resistance/Excitement	1.5	157.934	**.001**	.410	1	10.627	**.001**	.045	1.5	16.594	**.001**	.068
PANSS Total score	1.7	322.633	**.001**	.590	1	74.699	**.001**	.250	1.7	13.796	**.001**	.058
GAF score	2	174.899	**.001**	.500	1	3.954	**.048**	.020	2	4.478	**.013**	.025
HoNOS Behavioral problems	1.6	184.226	**.001**	.466	1	3.532	.061	.015	1.6	5.570	**.007**	.024
HoNOS Impairment	1.5	203.404	**.001**	.335	1	106.865	**.001**	.318	1.5	20.376	**.001**	.082
HoNOS Psychiatric symptoms	1.7	234.638	**.001**	.505	1	18.275	**.001**	.074	1.7	3.038	.057	.013
HoNOS Social problems	1.6	183.969	**.001**	.444	1	80.302	**.001**	.259	1.6	9.461	**.001**	.040

Variable	EMM (SE)	
	FEP/PAUSS-	FEP/PAUSS +
T0 PANSS Positive symptoms	17.060 (.501)	18.025 (.691)
T1 PANSS Positive symptoms	10.907 (.496)	13.316 (.684)
T2 PANSS Positive symptoms	9.693 (.406)	11.228 (.559)
T0 PANSS Negative symptoms	19.537 (.465)	31.633 (.639)
T1 PANSS Negative symptoms	15.369 (.572)	24.848 (.786)
T2 PANSS Negative symptoms	14.201 (.622)	20.456 (.854)
T0 PANSS Disorganization	17.568 (.456)	28.924 (.624)
T1 PANSS Disorganization	13.453 (.469)	21.532 (.642)
T2 PANSS Disorganization	12.642 (.473)	17.924 (.648)
T0 PANSS Affect	15.227 (.434)	16.949 (.598)
T1 PANSS Affect	11.007 (.395)	12.772 (.544)
T2 PANSS Affect	10.127 (.378)	11.127 (522)
T0 PANSS Resistance/Excitement	9.387 (.399)	12.620 (.550)
T1 PANSS Resistance/Excitement	7.280 (.345)	8.949 (.476)
T2 PANSS Resistance/Excitement	6.167 (.308)	6.278 (.425)
T0 PANSS Total score	82.136 (1.693)	111.342 (2.235)
T1 PANSS Total score	60.007 (1.910)	83.873 (2.606)
T2 PANSS Total score	54.653 (1.893)	69.215 (2.582)
T0 GAF score	44.159 (.874)	43.392 (1.373)
T1 GAF score	59.881 (1.012)	53.667 (1.591)
T2 GAF score	63.540 (1.025)	62.843 (1.611)
T0 HoNOS Behavioral problems	3.391 (.194)	4.275 (.267)
T1 HoNOS Behavioral problems	1.629 (.160)	2.125 (.220)
T2 HoNOS Behavioral problems	1.159 (.145)	1.075 (.199)
T0 HoNOS Impairment	2.060 (.139)	4.462 (.191)
T1 HoNOS Impairment	1.232 (.116)	2.913 (.160)
T2 HoNOS Impairment	1.126 (.110)	2.100 (.151)
T0 HoNOS Psychiatric symptoms	10.118 (.276)	11.150 (.381)
T1 HoNOS Psychiatric symptoms	5.717 (.288)	7.975 (.397)
T2 HoNOS Psychiatric symptoms	4.533 (.262)	5.913 (.361)
T0 HoNOS Social problems	6.138 (.272)	10.200 (.375)
T1 HoNOS Social problems	3.862 (.244)	6.688 (.336)
T2 HoNOS Social problems	3.158 (.219)	5.348 (.302)

As all Mauchly’s tests of sphericity are statistically significant (p < 0.05), Greenhouse–Geisser corrected degrees of freedom to assess the significance of the corresponding F value are used. Statistically significant p values are in bold. Statistical trends in p value (p < 0.01) are underlined. Bonferroni’s corrected p values are reported. Statistically significant p values are in bold

*ANOVA*  analysis of variance, *PAUSS* PANSS Autism severity scores, *FEP/PAUSS +* FEP patients with PAUSS score of > 30, *FEP/PAUSS-* FEP patients without PAUSS score of > 30, *PANSS*  Positive and negative syndrome scale, *df* degrees of freedom, *F* F statistic value, *GAF* Global assessment of functioning, *HoNOS* Health of the nation outcome scale, *p* statistical significance, *η2* partial eta squared, *EMM* Estimated marginal mean, *SE* Standard error, *T0* baseline assessment, *T1* 1-year assessment time, *T2* 2-year assessment time

Moreover, statistically significant interaction (time x group) effects were also found for PANSS total scores, PANSS “Negative Symptoms”, “Disorganization”, and “Resistance/Excitement-Activity” factor subscores, GAF scores, and HoNOS “Behavioral Problems”, “Impairment”, and “Social Problems” domain subscores. Specifically, in comparison with FEP/PAUSS-, FEP/PAUSS + subjects showed better longitudinal improvements in all these clinical dimensions (for details, see Table 4 and Figure S2 in the Supplementary Materials).

Across the follow-up, a statistically relevant decrease in PAUSS scores was observed both in the FEP total group and the FEP/PAUSS + subgroup (Table 5). Our multiple linear regression analysis results also showed that lower T1 equivalent dose of fluoxetine and higher total number of case management sessions significantly predicted improvements in the delta between T0 and.

**Table 5:** Longitudinal comparisons on PAUSS scores in the FEP total group and in the FEP/PAUSS + subgroup across the 2-year follow-up period

Variable	T0 (n = 301)	T1 (n = 276)	T2 (n = 229)	T0-T1 z (p)	T1-T2 z (p)	T0-T2 z (p)
PAUSS scores	24.99 ± 9.87	20.02 ± 8.95	17.35 ± 8.73	− 10.331 (**.001**)	− 7.433 (**.001**)	− 10.932 (**.001**)
Variable	T0 (n = 85)	T1 (n = 81)	T2 (n = 79)	T0-T1 z (p)	T1-T2 z (p)	T0-T2 z (p)
PAUSS scores	37.83 ± 4.58	28.08 ± 8.31	22.53 ± 9.01	− 7.320 (**.001**)	− 5.910 (**.001**)	− 7.618 (**.001**)

Mean ± standard deviation and Wilcoxon test (z) values are reported. Bonferroni’s corrected p values are reported. Statistically significant p values are in bold

*PAUSS* PANSS Autism Severity Scores, *FEP* First Episode Psychosis, *FEP/PAUSS +*  FEP patients with PAUSS score of > 30, *T0* baseline assessment time, *T1* 1-year assessment time, *T2* 2-year assessment time, *p* statistical significance

T2 PAUSS scores in our FEP total population (Table 6).

**Table 6:** Linear regression analysis results for T0-T2 delta PAUSS score by specialized treatment components of the Pr-EP program in the FEP total group across the 2-year follow-up period

T0–T2 PAUSS (n = 229)	B	SE	Β	p	95% CI lower upper		R2 = .061 F [df = 6] = 2.362 p = **.031**
Constant T0 equivalent dose of risperidone (mg/day) T1 equivalent dose of risperidone (mg/day) T2 equivalent dose of risperidone (mg/day) T2 number of individual psychotherapy sessions T2 number of family psychoeducation sessions T2 number of case management sessions	7.241 .201 − .651 .012 .029 .019 .053	1.491 .239 .255 .088 .047 .087 .020	− .067 − .214 .009 .045 .016 .187	.001 .402 **.011** .893 .533 .826 **.009**	4.303 − .271 − 1.154 − .162 − .063 − .152 .013	10.178 .672 − .148 .186 .122 .191 .093	

Statistically significant p values are in bold

*PAUSS* PANSS Autism severity scores, *FEP* First episode psychosis, *FEP/PAUSS +*  FEP patients with PAUSS score of > 30, *Pr-EP* Parma early psychosis, *T0* baseline assessment time, *T2* 2-year assessment time, *APS* Attenuated psychotic symptoms, *B* = unstandardized coefficient, *SE* Standard error, *β* standardized coefficient, *p* statistical significance, Bonferroni corrected p values are reported, *95% CI* 95% Confidence Interval for B, *R2* R-squared or coefficient of determination, *F* statistic test value for linear regression analysis

## Discussion

In recent years, the PAUSS has become a popular measure of autistic characteristics in patients with psychosis, including FEP [67]. Indeed, it is quickly and easily calculated from the PANSS, a widely used instrument for the assessment of psychosis psychopathology. Although initial validation of its psychometric properties in different studies was encouraging [68], evidence on PAUSS longitudinal course and stability remain poor and conflicting [15], especially in young people with FEP. This investigation contributed to fill this knowledge gap, particularly exploring PAUSS long-term stability and its relevant associations with outcome variables, clinical parameters and treatment response. This could lead to clarify the real psychopathological meaning of the PAUSS in patients with psychosis, as well as its prospective clinical relevance in FEP subjects treated within a specialized EIP service.

In the current investigation, about *a third* of FEP participants at presentation scored above the PAUSS cut-off of 30 and was included in the FEP/PAUSS + subgroup. This finding fell in the middle range of the wide PAUSS + prevalence rate in psychosis reported in the literature to date, varying from 1.5 to 50%. These discrepancies among studies are also probably due to important differences in PAUSS cut-offs and samples’ clinical characteristics (e.g., FEP vs SSD populations, early phase vs prolonged psychosis). Indeed, some authors used the original *PAUSS cut-off* based on the last percentile of score distribution described in its validation study [24], while others derived their cut-off from PAUSS median score [23] or alternative values optimizing the sensitivity/specificity ratio through “Receiver Operating Characteristics” (ROC) analyses [28]. In this respect, a score of ≥ 24 in patients with prolonged SSD [20] was detected to predict DSM-IV-TR ASD diagnosis. In our FEP population, we empirically found an optimal PAUSS cut-off score of ≥ 27 for predicting participants scored above the Autism Quotient (AQ) cut-off [69] (see Supplementary Materials [Table S2 and Figure S3] for details). However, further research aiming to face these psychometric issues and to identify the best cut-offs to use in different psychosis populations is needed. In particular, multicentric investigations and large, more homogeneous samples should be preferred. In this research, we used the original PAUSS cut-off score to allow results’ comparison with other similar clinical investigations.

Compared to FEP/PAUSS- at entry, our FEP/PAUSS + individuals showed higher prevalence rate of *SSD diagnosis* and grater *severity in* psychosis *psychopathology*, especially in terms of negative symptoms, disorganization, cognitive impairment, and behavioral problems (i.e., poor impulse control, hyperactivity, and aggressiveness), as well as higher baseline levels of *uncooperativeness*. This partly supports what was previously reported in other comparable, cross-sectional FEP studies. In this respect, Davut and co-workers [17] found an inverse baseline association between autistic symptom severity (measured with the PAUSS) and impairment in executive functions in 60 Turkish patients with FEP. Jeong and colleagues [22] observed that cognitive deficits increased with the severity of autistic features in a sample of 671 young Korean individuals with recent-onset psychosis. Overall, these findings substantially confirm the close link between autistic-like characteristics (evaluated with the PAUSS) and multiple cognitive domains abnormalities reported also in patients with SSD [23, 70–72].

Otherwise, more conflicting is the evidence on baseline PANSS total score, with some authors reporting no inter-group difference [24], while others suggesting higher severity levels in psychosis psychopathology in the PAUSS + subgroup [20, 23]. When interpreting these results, it should however be emphasized the potential influence of higher prevalence rate of SSD in our FEP/PAUSS + sample. Indeed, the PAUSS derives from the PANSS, a scale specifically developed for assessing psychopathology in schizophrenia [73], and therefore more sensitive in clinically defining the symptoms of SSD rather than non-SSD psychoses. This can also partly justify both higher baseline levels of uncooperativeness and lower antidepressant prescription rate at presentation in our FEP/PAUSS + subjects.

Moreover, the FEP/PAUSS + subgroup showed higher levels of *social problems*, both in terms of difficulty in interpersonal relationships and deficits in daily activities, housing/life condition, and employment. This supports the idea that higher PAUSS values at presentation are indices of worse socio-occupational functioning [17, 22, 25] and lower social skills [16] in young FEP individuals, similarly to subjects with prolonged SSD [20, 24]. The presence of more serious social problems can also justify the higher rate of case management interventions offered at entry to the FEP/PAUSS + subgroup within the Pr-EP program.

No baseline between-group difference in terms of sociodemographic and other clinical features was found. On the contrary, other authors reported lower levels of quality of life [15], higher prevalence of males [16, 17], and fewer years of education [17] in FEP participants scoring above the PAUSS cut-off score.

As for *outcome* parameters, our FEP/PAUSS + participants showed *lower* incidence rates of *symptomatic remission* and *functional remission* over time compared to FEP/PAUSS- ones. These findings are in line with what was recently reported in the Korean Early Psychosis Cohort Study (KEPS), suggesting that at the 2-year follow-up, the baseline FEP/PAUSS + subgroup was identified as a relevant individual predictor for not achieving both symptomatic and functional remission (measured with the PANSS and the GAF, respectively) [25].

However, in the present examination, we notably found a lower 2-year incidence rate of *service disengagement* in our FEP/PAUSS + individuals in comparison with the FEP/PAUSS- subsample. This suggests a significant tendency in Pr-EP team members to offer stronger and more temporally stable retention in care to FEP/PAUSS + subjects. This clinical habit could be related to the higher prevalence rate of SSD at entry in the FEP/PAUSS + individuals. Indeed, mental health professionals usually consider this diagnosis as a major psychiatric condition typically characterized by diagnostic stability [74], prolonged course, poor prognosis, and therefore mostly requiring enduring (sometimes uninterrupted) interventions [75].

Furthermore, in partial agreement with the results reported in the KEPS study [22], we found statistically relevant *group effects* in longitudinal improvements of GAF, HoNOS, and PANSS scores. Specifically, compared to FEP/PAUSS-, our FEP/PAUSS + participants had worse outcomes in terms of both psychopathological severity levels and daily functioning (i.e., they had higher scores across the 2 years of follow-up). However, in comparison with FEP/PAUSS-, FEP/PAUSS + subjects notably showed a significant interaction (“time x group”) effect in terms of better improvement in severity levels of psychopathology (especially in terms of negative symptoms, disorganization, resistance and excitement-activity), behavioral problems, daily and social functioning across the 2-year follow-up period (i.e., they had higher reduction in their scores at the end of our observation). In this sense, despite their worst outcome scores, our FEP/PAUSS + participants still showed a good treatment response within the Pr-EP program. Together with the stronger retention in care observed in our FEP/PAUSS + participants, these findings also suggest that counteracting service disengagement and favoring care continuity overtime are helpful in improving their functioning, clinical outcomes, and prognosis.

Finally, across the follow-up, a statistically relevant decrease in PAUSS scores was observed (both in the FEP total group and the FEP/PAUSS + subsample). This result does *not* support the *long-term stability* in PAUSS severity levels. According to Chisholm and colleagues [28], these longitudinal changes could be influenced by state-like psychotic symptoms rather than autistic traits. Therefore, in contrast to the theoretical philosophy behind the PAUSS (i.e., that some PANSS items/features are indicative of an “autistic phenotype” and autistic traits) [20], our findings support the idea that the PAUSS more probably represents a proxy for psychotic symptom severity (especially negative and disorganized symptoms) rather than autistic characteristics. In other words, the PAUSS seems to more reliably assess some clinical aspects of FEP psychopathology, probably detecting a distinct FEP subgroup with greater symptom severity and poorer outcomes, but it wouldn’t truly capture autism.

Moreover, in our total FEP population, this PAUSS longitudinal reduction appeared to be specifically predicted by a higher total number of *case management sessions* offered during the 2 years of the Pr-EP program, together with lower antidepressant dosage. This suggests that specialized psychosocial interventions are very helpful in longitudinally promoting improvement of negative and disorganized features in FEP individuals.

### Limitations

Several limitations should also be acknowledged when examining the results of this research. First, our findings are exclusively generalizable to youths with FEP. Indeed, they are not comparable with subjects affected by prolonged psychosis. In this respect, samples discrepancies may partly lead to differences in results and interpretations among studies.

Another important limitation is related to the absence of a control group including individuals with ASD and subjects without psychosis, as well as to the relatively small sample size, especially in terms of FEP/PAUSS + patients. Future investigations including larger PAUSS + samples are therefore needed to confirm our findings.

Third, a relevant part (40%) of our FEP sample was composed of patients affected by psychotic disorders not belonging to SSD (i.e., affective psychosis, brief psychotic disorder, and psychotic disorder NOS) at entry. Although the PANSS has been commonly used in clinical populations of individuals with FEP (including the entire diagnostic spectrum of psychosis), the PAUSS was specifically examined exclusively in FEP individuals with schizophrenia or SSD. This diagnostic heterogeneity may affect the PAUSS stability in our investigation. Therefore, future studies examining the PAUSS in larger non-SSD FEP samples are needed.

Finally, another crucial weakness is that our FEP participants were recruited within only one EIP service. Thus, future multicenter investigations are absolutely needed.

## Conclusion

The PAUSS has recently become a popular measure to assess “autistic features” in current and archival datasets including FEP patients. However, the results of this examination showed no long-term stability in its scores, suggesting that it could not represent a valid instrument to detect autistic characteristics/profiles in youths with FEP. On the contrary, the PAUSS may capture psychotic symptom severity and functioning impairment rather than autism features, especially reflecting the negative/disorganization symptom load. In other words, being derived from the PANSS, it could represent a proxy for assessing the clinical severity of FEP rather than a “pure indicator” of autism characteristics.

Moreover, the PAUSS could also be used as a helpful baseline psychopathological index of poorer clinical and functional outcomes in FEP patients, although it seems to be sensitive to specific specialized psychosocial interventions offered within the Pr-EP program (especially case management). In this sense, the PAUSS should not replace an accurate clinical judgment and the administration of more appropriate tools evaluating ASD traits and diagnosis.

## Supplementary Information

Below is the link to the electronic supplementary material.

Supplementary file1 (DOCX 341 KB)

## Data Availability

The data that support the findings of this research are not publicly available due to privacy and/or ethical restrictions. However, the data are available on reasonable request from the corresponding author.
