# Supplementary material for: What autism features in first episode psychosis? Results from a 2-year follow-up study
Source: Eur Arch Psychiatry Clin Neurosci. 2025 Mar 5;275(8):2403–16. doi: 10.1007/s00406-025-01986-1 (PMC12638376; doi:10.1007/s00406-025-01986-1)
Supplement: Supplementary file 1 — Supplementary file1 (DOCX 341 KB) [file 406_2025_1986_MOESM1_ESM.docx]

Table S1 – PAUSS items from the PANSS.

| Autism behavioral clusters  (DSM-IV-TR) | PANSS items | Original PANSS descriptions |
| --- | --- | --- |
| *Deficits in social interaction*  *Deficits in communication*  *Restricted/repetitive patterns of behavior* | Blunted affect (N1)  Poor rapport (N3)  Passive/apathetic social withdrawal (N4)  Difficulty in abstract thinking (N5)  Lack of spontaneity/flow of conversation (N6)  Stereotyped thinking (N7)  Mannerisms and posturing (G5)  Preoccupation (G15) | “Diminished emotional responsiveness as characterized by a reduction in facial expression, modulation of feelings and communicative gestures”.  “Lack of interpersonal empathy, openness in conversation and sense of closeness, interest or involvement with the interviewer”.  “Diminished interest and initiative in social interactions due to passivity, apathy, anergy or avolition”.  “Impairment in the use of the abstract-symbolic mode of thinking”.  “Reduction in the normal flow of communication associated with apathy, avolition, defensiveness or cognitive deficit”.  “Decreased fluidity, spontaneity and flexibility of thinking, as evidenced in rigid, repetitious or barren thought content”.  “Unnatural movements or posture as characterized be an awkward, stilted, disorganized, or bizarre appearance”.  “Absorption with internally generated thoughts and feelings and with autistic experiences to the detriment of reality orientation and adaptive behavior”. |

Note – PAUSS = PANSS Autism Severity Score; PANSS = Positive And Negative Syndrome Scale; DSM-IV-TR = Diagnostic and Statistical Manual of mental disorders, IV Edition, Text Revised.

Figure S1 – Kalan-Meyer survival functions: comparisons on 2-year time-to-event outcome incidence rate among the two FEP subgroups.


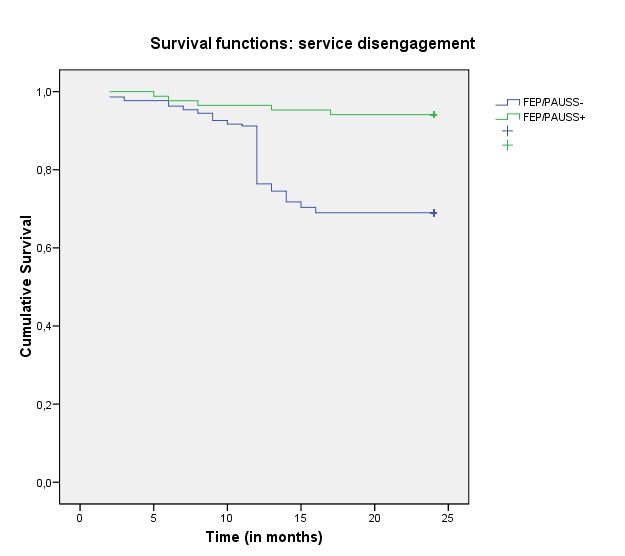


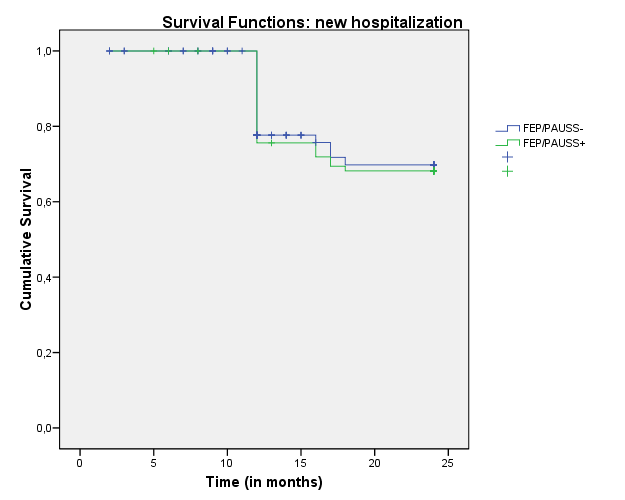


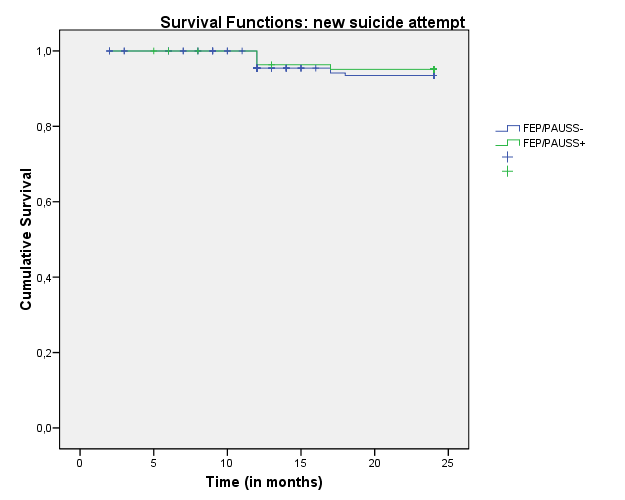


Note. FEP = First Episode Psychosis; PAUSS = PANSS Autism Severity Scores; FEP/PAUSS+ = FEP patients with PAUSS > 30; FEP/PAUSS- = FEP patients without PAUSS > 30.

Figure S2 – Profile plots: mixed ANOVA results on psychopathological and outcome parameters across the 2-year follow-up period in the two FEP subgroups.


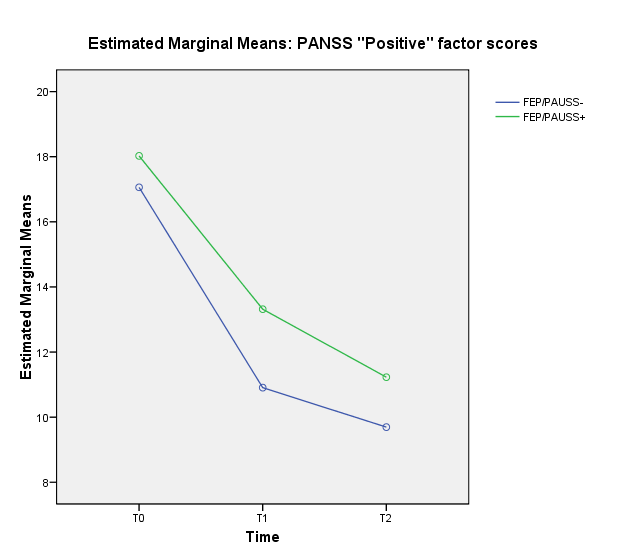


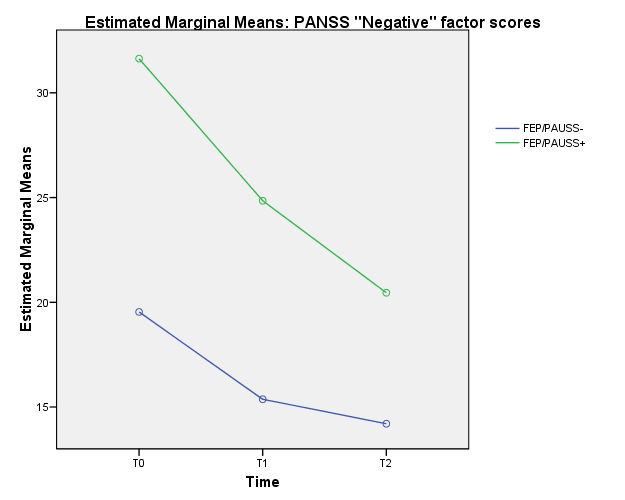


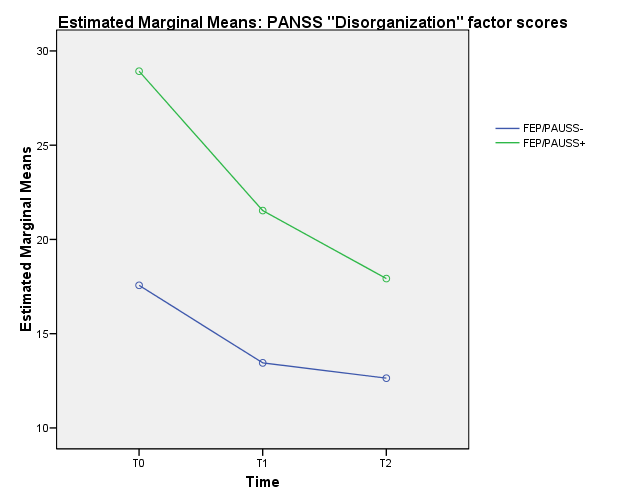


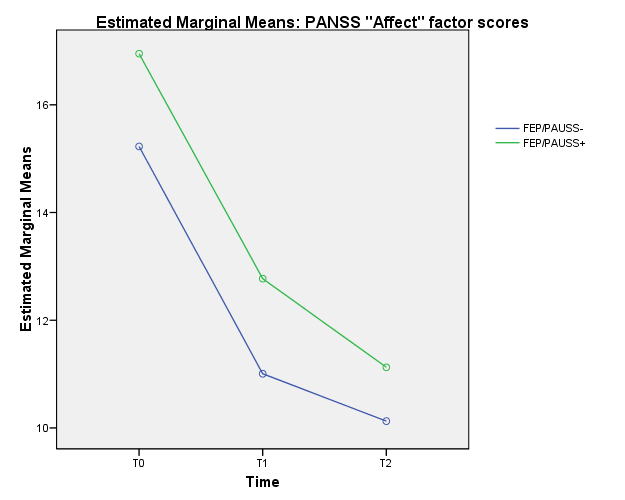


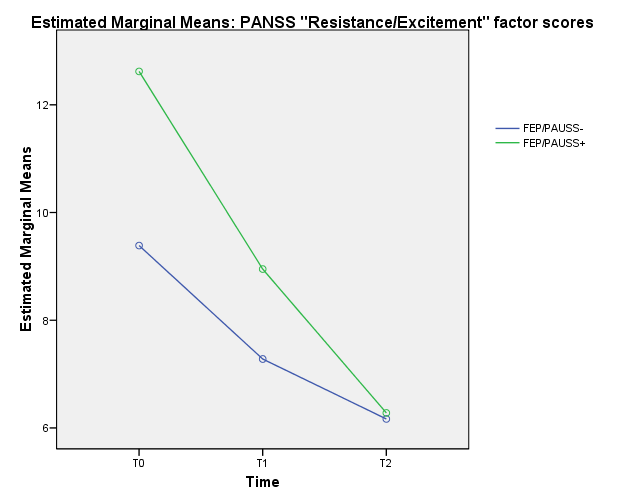


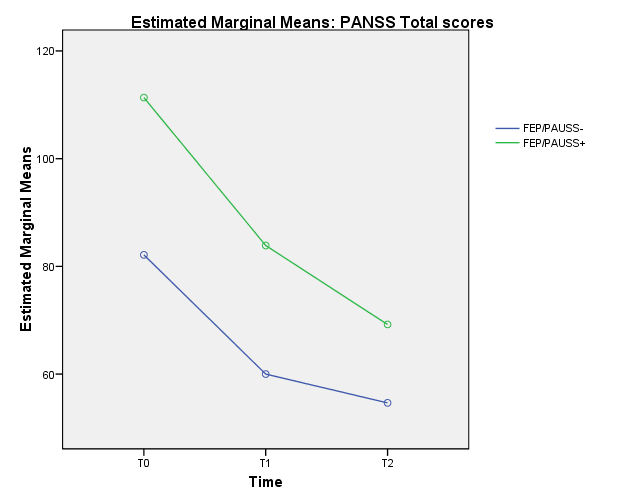


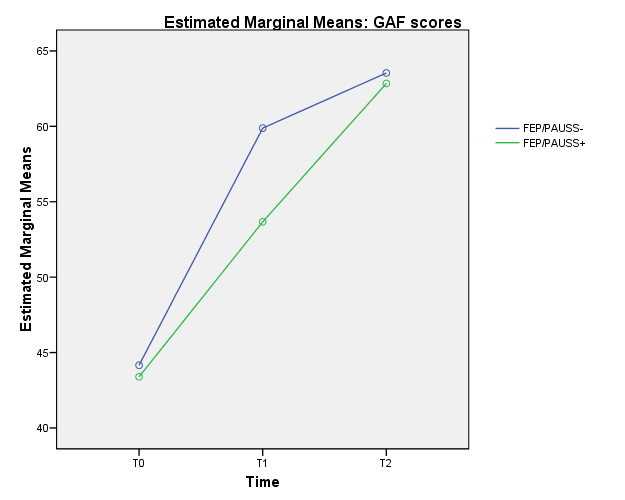


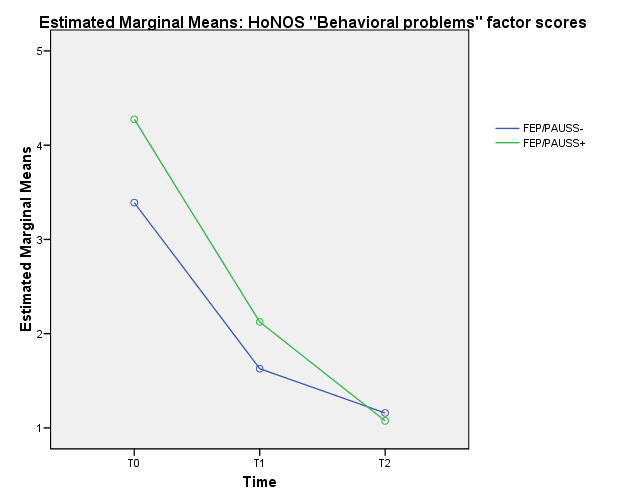


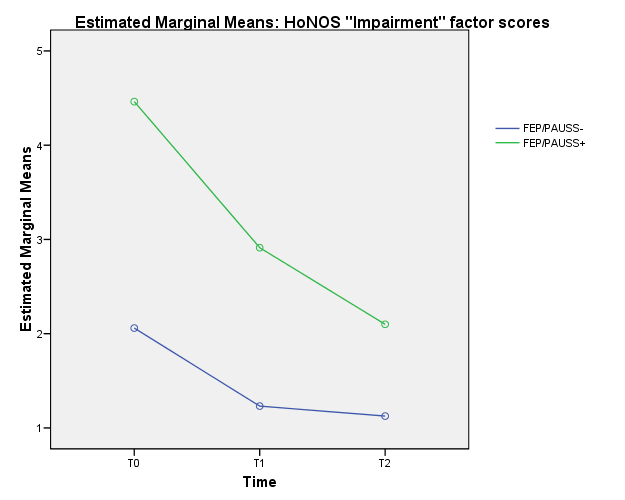


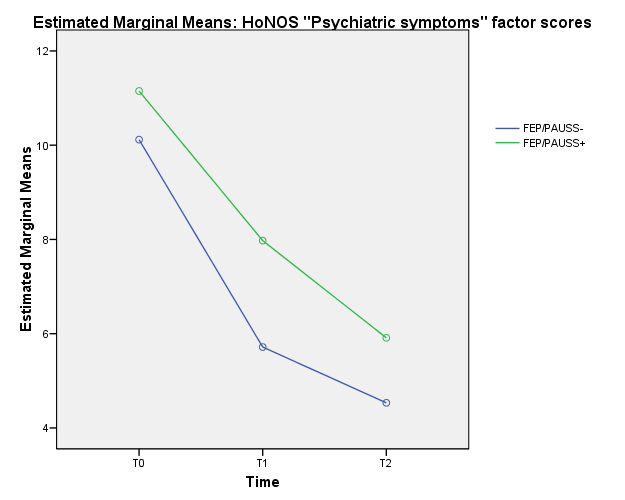


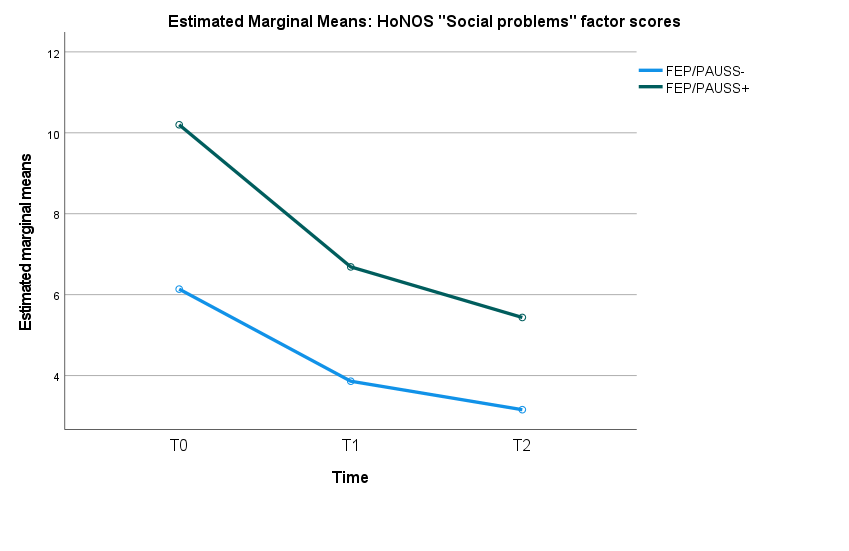


Note - ANOVA = analysis of variance; PANSS = Positive And Negative Syndrome Scale; PAUSS = PANSS Autism Severity Scores; FEP/PAUSS+ = FEP patients with PAUSS > 30; FEP/PAUSS- = FEP patients without PAUSS > 30; PANSS = Positive And Negative Syndrome Scale; GAF = Global Assessment of Functioning; T0 = baseline assessment; T1 = 1-year assessment time; T2 = 2-year assessment time.

Table S2 – AQ classification accuracy by PAUSS cut-off scores in both FEP total sample and the FEP/SSD subgroup at baseline.

| FEP total sample (n = 301) – AUC = .621 | | | | |
| --- | --- | --- | --- | --- |
| PAUSS  cut-off scores | Sensitivity | Specificity | d | J |
| > 29 | .357 | .923 | .647 | .280 |
| **> 27** | .429 | .874 | .560 | .304 |
| > 26 | .464 | .837 | .585 | 301 |
| > 19 | .643 | .529 | .591 | .172 |
| > 18 | .714 | .490 | .584 | .204 |
| > 15 | .750 | .356 | .691 | .106 |
| FEP/SSD subgroup (n = 170) – AUC = .607 | | | | |
| PAUSS  cut-off scores | Sensitivity | Specificity | d | J |
| > 29 | .438 | .894 | .572 | .332 |
| **> 27** | .500 | .851 | .522 | .351 |
| > 20 | .563 | .468 | .688 | .031 |
| > 19 | .688 | .447 | .634 | .135 |
| > 18 | .750 | .383 | .665 | .133 |
| > 15 | .813 | .340 | .685 | .153 |

Note - AQ = Autism spectrum Quotient; PAUSS = PANSS Autism Severity Score; FEP = First Episode Psychosis; FEP/SSD = FEP participants with Schizophrenia Spectrum Disorder; d = distance between the point (0,1) at each cut-off point on the ROC curve; J = Youden index. An AUC value of > .700 was acceptable. The optimal cut-offs are in bold.

Figure S3 - Receiver Operating Characteristics (ROC) curves of PAUSS scores predicting FEP/AQ+ vs FEP/AQ- subgroup at baseline in both FEP total sample and the FEP/SSD subgroup.


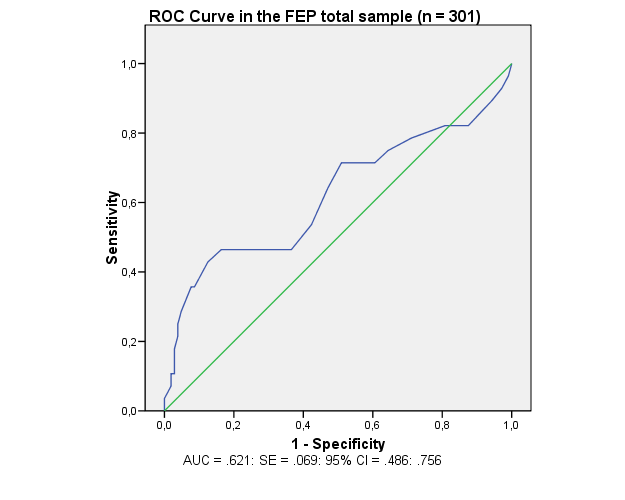


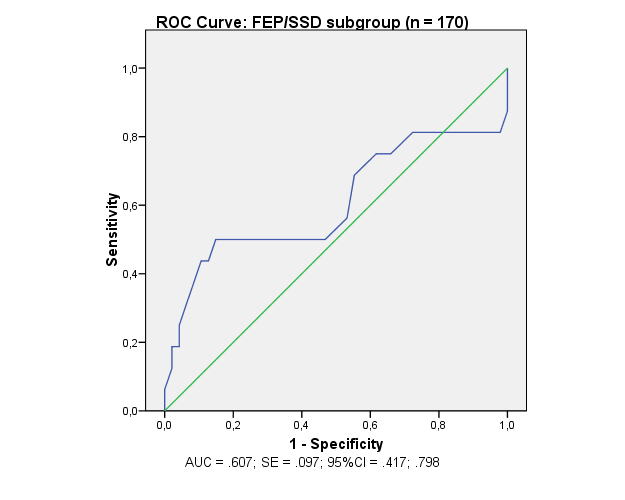


Note - AQ = Autism Quotient; PAUSS = PANSS Autism Severity Score; FEP = First Episode Psychosis; FEP/SSD = FEP participants with Schizophrenia Spectrum Disorder; FEP/AQ+ = FEP patients with AQ total score > 25 (presence of autistic traits); FEP/AQ- = FEP patients with AQ total score ≤ 25 (absence of autistic traits); ROC = Receiver Operating Characteristics; AUC = Area under the ROC curve; SE = Standard Error; CI = Confidence Interval. An AUC value of > .700 was acceptable.
